# Ischemic Stroke in a Child after a Probable Scorpion Sting: Correspondence

**DOI:** 10.4269/ajtmh.25-0536

**Published:** 2025-12-11

**Authors:** Santosh Govind Rathod, Diksha Sabharwal

**Affiliations:** Department of Clinical Hematology AIIMS Nagpur Maharashtra India E-mail: drsgrathod2007@gmail.com; Department of Internal Medicine Dr. D.Y. Patil College Hospital and Research Center Pune E-mail: dikshasab12@gmail.com

Dear Editor,

We read with great interest the article, *“Ischemic Stroke in a Child after a Probable Scorpion Sting”* by Laura Naranjo, et al.^1^ We aim to emphasize an important role of prazosin in managing severe scorpion stings. In the presented case, the clinician did not include prazosin in the management of a severe scorpion sting that resulted in autonomic storm in a 2-year-old male child (with pulmonary edema, cardiogenic shock, and ischemic stroke). Alfa toxin is a pathogenic scorpion toxin that inhibits the inactivation of neuronal sodium channels, resulting in a sustained depolarization and neuronal excitation, as well as the release of endogenous catecholamines, neuropeptide-Y, and endothelin-1.^2^ Heart failure in severe scorpion stings is caused primarily by persistent alpha-receptor activation, catecholamine-induced myocarditis, and neuropeptide-Y–induced myocardial ischemia.^2^ Endothelin-1 inhibits epithelial sodium channels, cystic-fibrosis transmembrane conductors, and Na+/K-ATPase channels in the lungs, causing refractory pulmonary edema.^3^

Prazosin is a selective alpha-1 blocker that lowers preload and left ventricular impedance without producing tachycardia and reverses the hemodynamic and metabolic effects of circulating catecholamines.^2^ Prazosin inhibits phosphodiesterase, hence increasing cellular cyclic GMP. An endothelin inhibitor reduces pulmonary edema and inhibits platelet aggregation.^2^ Prazosin neutralizes the effects of venom-liberated catecholamines but does not act on the venom itself. In severe scorpion stings, the bite site serves as a store of venom, which is progressively released into the bloodstream due to prolonged stimulation of the alpha-1 receptor. Prazosin, as a vasodilator, allows scorpion venom to be neutralized by antivenom^2^^,^^4^ Thus the simultaneous use of prazosin and antivenom appears to hasten clinical improvement and may decrease mortality.
